# Olanzapine, but not clozapine, increases glutamate release in the prefrontal cortex of freely moving mice by inhibiting D-aspartate oxidase activity

**DOI:** 10.1038/srep46288

**Published:** 2017-04-10

**Authors:** Silvia Sacchi, Vito De Novellis, Giovanna Paolone, Tommaso Nuzzo, Monica Iannotta, Carmela Belardo, Marta Squillace, Paolo Bolognesi, Elena Rosini, Zoraide Motta, Martina Frassineti, Alessandro Bertolino, Loredano Pollegioni, Michele Morari, Sabatino Maione, Francesco Errico, Alessandro Usiello

**Affiliations:** 1 Dipartimento di Biotecnologie e Scienze della Vita, Università degli studi dell’Insubria, 21100, Varese, Italy; 2 The Protein Factory, Politecnico di Milano and Università degli studi dell’Insubria, 20131, Milano, Italy; 3 Department of Experimental Medicine, Section of Pharmacology, The Second University of Naples (SUN), 80138, Naples, Italy; 4 Department of Medical Sciences, Section of Pharmacology, University of Ferrara and National Institute of Neuroscience, 44100, Ferrara, Italy; 5 Laboratory of Behavioural Neuroscience, Ceinge Biotecnologie Avanzate, 80145, Naples, Italy; 6 Department of Environmental, Biological and Pharmaceutical Sciences and Technologies, Second University of Naples (SUN), 81100, Caserta, Italy; 7 Department of Basic Medical Science, Neuroscience and Sense Organs, University of Bari Aldo Moro, 70121, Bari, Italy; 8 Department of Molecular Medicine and Medical Biotechnology, University of Naples “Federico II”, 80131, Naples, Italy

## Abstract

D-aspartate levels in the brain are regulated by the catabolic enzyme D-aspartate oxidase (DDO). D-aspartate activates NMDA receptors, and influences brain connectivity and behaviors relevant to schizophrenia in animal models. In addition, recent evidence reported a significant reduction of D-aspartate levels in the *post-mortem* brain of schizophrenia-affected patients, associated to higher DDO activity. In the present work, microdialysis experiments in freely moving mice revealed that exogenously administered D-aspartate efficiently cross the blood brain barrier and stimulates L-glutamate efflux in the prefrontal cortex (PFC). Consistently, D-aspartate was able to evoke L-glutamate release in a preparation of cortical synaptosomes through presynaptic stimulation of NMDA, mGlu5 and AMPA/kainate receptors. In support of a potential therapeutic relevance of D-aspartate metabolism in schizophrenia, *in vitro* enzymatic assays revealed that the second-generation antipsychotic olanzapine, differently to clozapine, chlorpromazine, haloperidol, bupropion, fluoxetine and amitriptyline, inhibits the human DDO activity. In line with *in vitro* evidence, chronic systemic administration of olanzapine induces a significant extracellular release of D-aspartate and L-glutamate in the PFC of freely moving mice, which is suppressed in *Ddo* knockout animals. These results suggest that the second-generation antipsychotic olanzapine, through the inhibition of DDO activity, increases L-glutamate release in the PFC of treated mice.

Free D-aspartate (D-Asp) levels are markedly high in the mammalian brain during embryonic and perinatal stages, before gradually dropping at adulthood^1^,^2^,^3^,^4^. The age-dependent changes in free D-Asp levels are regulated by the catabolic enzyme D-aspartate oxidase (DDO), whose transcription and activity rise from prenatal to postnatal phase^4^,^5^. Electrophysiological evidence in mouse brain showed that D-Asp activates N-methyl D-aspartate receptors (NMDARs) by binding to the glutamate (L-Glu) site of GluN2 subunits^6^,^7^,^8^,^9^. In agreement with the proposed neuromodulatory role of endogenous D-Asp, a recent study revealed that this D-amino acid can be detected in the extracellular space where it is released in a Ca^2+^-dependent manner^4^. Consistent with these findings, non-physiological high levels of D-Asp in *Ddo* knockout (*Ddo*^−/−^) and C57BL/6J mice chronically treated with this D-amino acid were associated with significant changes in NMDAR-dependent processes, including synaptic transmission, plasticity and cognition^6^,^7^,^8^,^9^,^10^,^11^. Besides its ability to stimulate postsynaptic NMDARs^12^,^13^, microdialysis experiments indicated that D-Asp can also increase L-Glu release in the prefrontal cortex (PFC) of freely moving mice^14^.

Worthy of note, in line with the hypo-glutamatergic hypothesis of schizophrenia^15^, preclinical studies documented that increased levels of free D-Asp in *Ddo*^−/−^ mice attenuate sensorimotor gating deficits induced by psychotomimetic drugs^9^,^16^, enhance cortico-hippocampal connectivity^16^, and prevent PCP-induced dysfunctional activation of brain circuits, as measured by functional magnetic resonance imaging (fMRI)^16^. In addition to studies in animal models, clinical investigation has shown that a single-nucleotide polymorphism in the *DDO* gene (rs3757351), predicting reduced expression of *DDO* mRNA in the *post-mortem* PFC, is associated in healthy humans to greater prefrontal gray matter and activity during working memory, as detected by fMRI^11^. Moreover, two independent studies recently showed a significant reduction (~30–40%) of total free D-Asp content in the *post-mortem* brain of schizophrenia-affected patients^17^,^18^. In particular, Nuzzo *et al*. reported that lower levels of D-Asp in the dorsolateral PFC (DLPFC) of patients with schizophrenia are associated to significantly increased DDO enzymatic activity, thus suggesting alterations in D-Asp metabolism in this psychiatric disorder^18^.

Even though the previous evidence has convincingly shown an influence of D-Asp on glutamatergic neurotransmission, many issues concerning the neurophysiology of this D-amino acid have still to be addressed. In the present work, we explored the ability of exogenous D-Asp to cross the blood brain barrier and assessed the mechanism by which this D-amino acid triggers the cortical release of L-Glu. On the other hand, it is still unclear whether the altered D-Asp metabolism observed in patients with schizophrenia is a pathophysiological trait or, rather, an epiphenomenon due to the chronicity of disease or to pharmacological treatment. This point is of particular clinical interest given that some of the second-generation antipsychotic drugs, like clozapine and olanzapine, are reported to affect not only dopaminergic and serotonergic neurotransmission but also the homeostasis of glutamatergic system^19^,^20^,^21^,^22^. Based on this consideration and on the ability of D-Asp to affect glutamatergic system^12^,^13^, in the second part of this work, we performed an *in vitro* screening of widely used first and second-generation antipsychotics to test their effect on DDO enzymatic activity. Among these compounds, olanzapine proved to be the only DDO inhibitor and was selected for further evaluating its ability to affect the *in vivo* release of D-Asp and L-Glu in the PFC of freely moving mice.

## Results

### Extracellular D-aspartate levels in the prefrontal cortex of freely moving mice rise after both chronic and acute treatment with this D-amino acid

**Figure 1:**
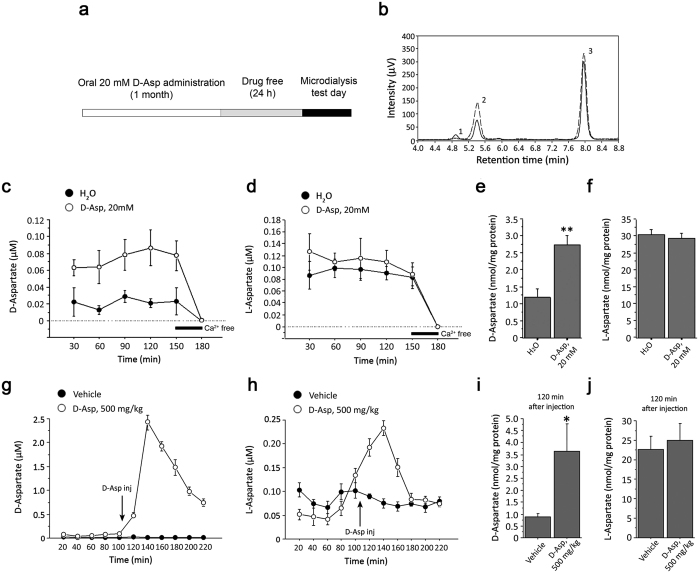
D-aspartate extracellular concentration in the mouse prefrontal cortex increases after chronic and acute administration of the D-amino acid. (**a**) Schematic timeline of the oral chronic D-aspartate administration procedure and microdialysis. (**b**) Examples of HPLC chromatograms illustrating the detection of D-Asp (1), L-Asp (2) and L-Glu (3) in a perfusate collected from the prefrontal cortex (PFC) of a mouse subjected to 20 mM D-Asp treatment. Retention times: D-Asp = 4.8 ± 0.1 min; L-Asp = 5.5 ± 0.1 min, L-Glu = 8.0 ± 0.2 (mean vaues ± SD; n = 10). The identity of D-Asp peak and the peak area was determined by analyzing the sample upon a pre-column treatment with beef DDO (grey dashed line). (**c**,**d**) Time course of extracellular concentration of free (**c**) D-Asp and (**d**) L-Asp in the PFC of mice chronically treated with 20 mM D-Asp and in their untreated controls (n = 5 per treatment). Last fraction of dialysates (150–180 min) was collected in a Ca^2+^-free ACSF. (**e**) Free D-Asp and (**f**) L-Asp total contents in PFC homogenates of chronically treated mice and controls (n = 4 per treatment). (**g**,**h**) Time course of extracellular concentration of free (**g**) D-Asp and (**h**) L-Asp in the PFC of mice subjected to the acute i.p. administration of 500 mg/kg D-Asp and in vehicle-treated animals (n = 5 per treatment). (**i**) Free D-Asp and (**j**) L-Asp total contents in PFC homogenates of acutely D-Asp-treated mice, after 2 h from treatment, and in vehicle-treated controls (n = 4 per treatment). The amount of free D-Asp and L-Asp in tissue homogenates was normalized by the total protein content of each sample. The graphs display the mean values ± SEM. *P < 0.05, compared to vehicle-treated mice; **P < 0.01, compared to untreated mice (Student’s t test).

We have previously demonstrated that 4-week oral treatment with 20 mM D-Asp enhances basal cerebral activity, spine density, dendritic length, and late-phase long-term potentiation in mice^11^. To quantify the extracellular content of D-Asp (and of its enantiomer, L-Asp) under this treatment regimen, we performed microdialysis in the PFC of freely moving C57BL/6J mice (Fig. 1a). Free Asp enantiomers were resolved by HPLC as single defined peaks with a retention time of 4.8 ± 0.1 min for D-Asp and 5.5 ± 0.1 min for L-Asp, while the peak corresponding to free L-Glu was detected at 8.0 ± 0.2 min (Fig. 1b). The identity of the observed peaks was verified by comparing the retention times of external standards and by selective enzymatic degradation (Fig. 1b). Microdialysis experiments confirmed that nanomolar concentrations of D-Asp are detectable in the cortical extracellular fluid of untreated freely moving C57BL/6J mice (Fig. 1c). Interestingly, two-way ANOVA with repeated measures revealed significantly increased cortical D-Asp extracellular levels in response to chronic treatment (F_(1,32)_ = 11.045, *P* = 0.0105; Fig. 1c). Indeed, a ~3.5-fold increase in extracellular D-Asp concentration was detected in treated animals compared to controls (average values: H_2_0 vs. chronic D-Asp, 21 ± 5 vs. 74 ± 8 nM). At the same time, no difference in cortical levels of L-Asp was evident between groups (F_(1,32)_ = 0.614, *P* = 0.4558; Fig. 1d). To evaluate whether the release of D-Asp and L-Asp was Ca^2+^-dependent, we collected the last dialysate fraction (150–180 min) by perfusing with Ca^2+^-free ACSF (Fig. 1c,d). Remarkably, the removal of Ca^2+^ from ACSF reduced D-Asp and L-Asp extracellular concentrations below the detection limit. In line with the higher extracellular levels of D-Asp, we also found a significant increase of total D-Asp content in homogenates of the contralateral PFC of treated mice, compared to untreated littermates (H_2_O vs. chronic D-Asp: 1.20 ± 0.26 vs. 2.76 ± 0.27 nmol/mg prot; *P* = 0.0057, Student’s t test; Fig. 1e). Conversely, no variation in total L-Asp content was apparent in the same animal groups (H_2_O vs. chronic D-Asp: 30.7 ± 1.5 vs. 29.4 ± 1.5 nmol/mg prot; *P* = 0.5759; Fig. 1f).

We then investigated the effect of acute D-Asp administration on the cortical extracellular levels of this D-amino acid. We used a dose of 500 mg/kg (intraperitoneal, i.p.) D-Asp since we previously found that this concentration elicited NMDAR-mediated signaling responses *in vivo*^9^. Remarkably, this treatment resulted in a rapid and robust increase of extracellular D-Asp levels in the PFC of freely moving C57BL/6J mice (F_(1,40)_ = 253.165; *P* < 0.0001; Fig. 1g). In particular, we observed a strong increase of D-Asp already 20 min after injection (470 ± 60 nM, ~47-fold increase over basal levels), a peak at 40 min (2430 ± 170 nM, ~240-fold increase) and a slow decrease thereafter (740 ± 80 nM at 120 min post-injection, Fig. 1g). Interestingly, acute D-Asp treatment also augmented L-Asp extracellular concentration (F_(1,40)_ = 53.987; *P* < 0.0001; Fig. 1h) although such increase (230 ± 10 nM at 40 min post-injection, ~3-fold increase over basal levels) was smaller compared to that of its D-enantiomer. Differently to D-Asp, L-Asp levels returned to baseline already 80 min after injection (Fig. 1h). To investigate whether the changes in D-Asp and L-Asp extracellular levels were also detected in brain extracts, animals subjected to microdialysis were sacrificed 2 h after injections and the contralateral PFC dissected for HPLC analysis of tissue homogenates. In line with the observed long-lasting increased extracellular D-Asp levels, we found a significant elevation of this D-amino acid in PFC homogenates from treated mice, compared to vehicle-treated group (vehicle vs. acute D-Asp: 0.88 ± 0.14 vs. 3.65 ± 1.13 nmol/mg prot; *P* = 0.0289; Fig. 1i). Conversely, no difference in the total content of the L-enantiomer was detected between acute D-Asp- and vehicle-treated groups (25.00 ± 4.39 vs. 22.65 ± 3.44 nmol/mg prot, respectively; *P* = 0.6809; Fig. 1j).

### D-Aspartate stimulates cortical L-glutamate release through presynaptic NMDA and non-NMDA ionotropic and metabotropic receptors

**Figure 2:**
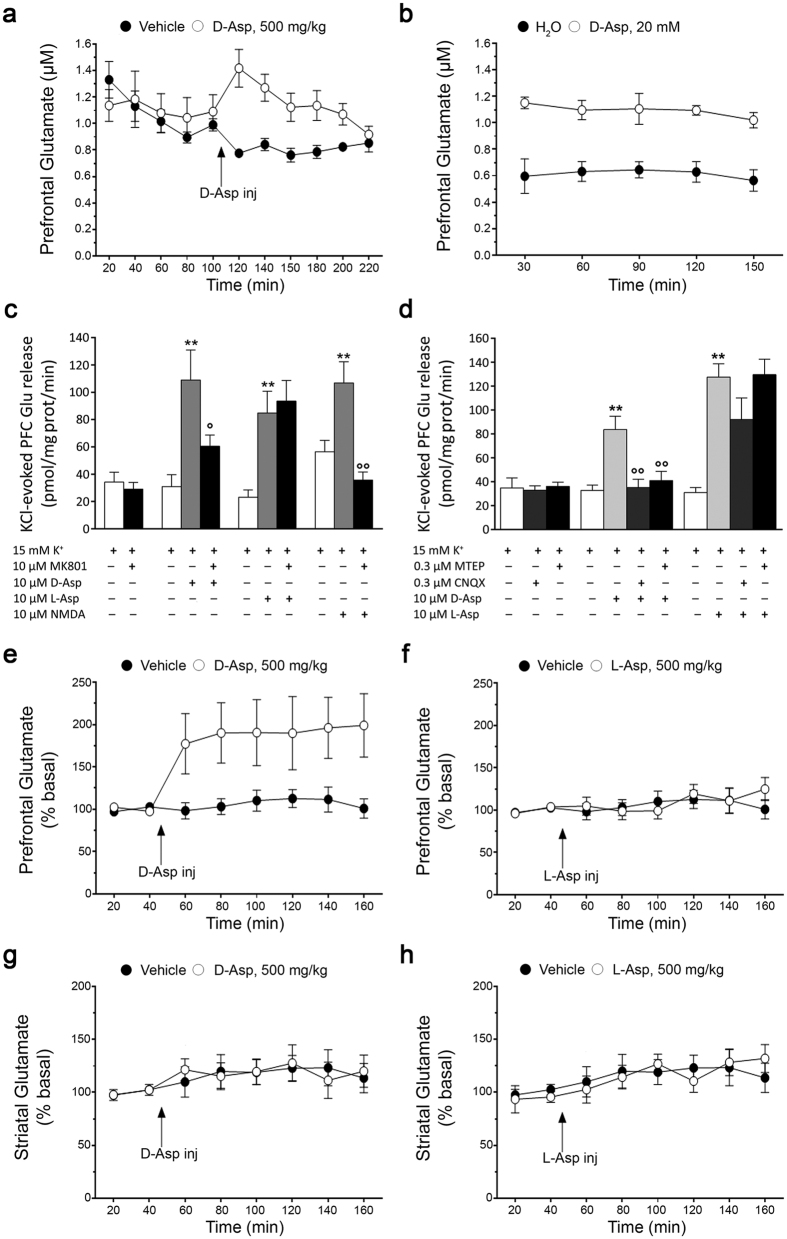
D-aspartate administration stimulates extracellular L-glutamate efflux through presynaptic NMDA and non-NMDA ionotropic and metabotropic receptors. (**a**,**b**) Time course of extracellular L-Glu levels in the prefrontal cortex (PFC) of mice (**a**) subjected to the acute administration of 500 mg/kg D-Asp or (**b**) chronically treated with 20 mM D-Asp, and their respective controls (n = 5 per treatment). The graphs displayed the mean values ± SEM. (**c**,**d**) Detection of KCl-evoked extracellular L-Glu release from cerebrocortical synaptosomes. Synaptosomes were continuously perfused with a medium containing the excitatory amino acids transporter inhibitor TBOA (10 μM), and stimulated with 15 mM K^+^ for 90 sec. D-Asp, L-Asp and NMDA (10 μM) were added 3 min prior K^+^ and maintained for further 3 min. (**c**) MK801, (**d**) MTEP or CNQX were added 3 min before agonists and maintained until the end of experiment. Data represent net extra release (i.e. release above baseline), and are expressed as mean ± SEM pmol/mg prot/min of the following numbers of determinations: (**c**) n = 8–10 (K^+^), n = 12–13 (D-Asp), n = 11–15 (L-Asp), n = 5–7 (NMDA); (**d**) n = 8–9 (K^+^), n = 12–18 (D-Asp), n = 9–12 (L-Asp). **P < 0.01, compared to K^+^ alone; °*P* < 0.05, °°*P* < 0.01, compared to K^+^ in the presence of agonist (one-way ANOVA followed by the Newman-Keuls test for multiple comparisons). (**e**–**h**) Time course of extracellular L-Glu levels in the (**e**,**f**) PFC (n = 12 vehicle, n = 9 D-Asp, n = 10 L-Asp) and (**g**,**h**) striatum (n = 9 vehicle, n = 11 D-Asp, n = 9 L-Asp) of mice after acute administration of (**e**,**g**) 500 mg/kg D-Asp or (**f**,**h**) 500 mg/kg L-Asp. The graphs display the mean values ± SEM.

We have previously reported that acute treatment with D-Asp increases extracellular L-Glu in the PFC of freely moving mice^14^. Here, we confirmed that acute treatment with 500 mg/kg D-Asp resulted in increased L-Glu efflux in the mouse PFC (two-way ANOVA with repeated measures: F_(1,40)_ = 25.578, *P* = 0.001; 20 min after injection: vehicle vs. acute D-Asp: 0.78 ± 0.03 vs. 1.42 ± 0.14 μM; Fig. 2a). Then we explored whether also chronic oral administration of D-Asp influences L-Glu release in the same brain region. Similarly to acute injection, we observed that one-month oral treatment with 20 mM D-Asp significantly increased extracellular L-Glu levels in the PFC (F_(1,32)_ = 34.454, *P* = 0.0004; Fig. 2b). Indeed, a ~2-fold increase of L-Glu concentrations was detected in the cortical dialysates of chronically treated mice, compared to untreated group (average values: H_2_O vs. chronic D-Asp, 0.61 ± 0.04 vs. 1.09 ± 0.03 μM; Fig. 2b).

These results prompted us to investigate the mechanism by which increased D-Asp levels stimulate L-Glu efflux from cortical glutamatergic nerve terminals. To this aim, the effect of D-Asp was tested in a preparation of cerebrocortical synaptosomes continuously superfused with a medium containing the non-selective excitatory amino acid transporter (EAAT) inhibitor TBOA (10 μM) (see Supplementary Methods). Spontaneous L-Glu levels were 21.3 ± 1.1 nM (n = 180). A 90 s pulse with 15 mM K^+^ caused a transient elevation (~2.5-fold) of L-Glu levels over basal values (time-course not shown), corresponding to a net release of 35.6 ± 3.4 pmol/mg prot/min (Fig. 2c). D-Asp, L-Asp and NMDA (perfused at 10 μM) caused similar elevations (up to ~3-fold) of the K^+^-evoked L-Glu release (112.1 ± 12.4, 86.9 ± 10.7 and 106.9 ± 15.4 pmol/mg prot/min, n = 30, n = 27, n = 6, respectively; *P* < 0.001; Fig. 2c). Interestingly, the non-competitive NMDAR antagonist MK801 fully prevented the stimulation evoked by NMDA (treatment effect: F_(2,15)_ = 12.61, *P* = 0.0006; NMDA vs. NMDA + MK801, *P* < 0.01), attenuated that evoked by D-Asp (treatment effect: F_(2,26)_ = 6.32, *P* = 0.0058; D-Asp vs. D-Asp + MK801, *P* < 0.05) but left unchanged the response to L-Asp (treatment effect: F_(2,34)_ = 6.97, *P* = 0.0029; L-Asp vs. L-Asp + MK801, *P* > 0.05; Fig. 2c). Moreover, AMPAR antagonist (CNQX) and mGluR5 antagonist (MTEP) prevented the effect of D-Asp (treatment effect: F_(3,56)_ = 9.84, *P* < 0.0001; D-Asp vs. D-Asp + CNQX or D-Asp + MTEP *P* < 0.01; Fig. 2d) but left unchanged that of L-Asp (treatment effect: F_(3,38)_ = 10.23, *P* < 0.0001; L-Asp vs. L-Asp + CNQX or L-Asp + MTEP *P* > 0.05), although a trend for inhibition was observed in the presence of CNQX (Fig. 2d).

Based on these observations, we investigated whether the systemic administration of L-Asp, as that of D-Asp, was able to trigger L-Glu release in the mouse PFC. Interestingly, microdialysis experiments showed a stereo-selectivity of D-Asp, compared to L-Asp, in the modulation of cortical L-Glu efflux. Indeed, the i.p. injection of 500 mg/kg D-Asp induced a persistent elevation of extracellular L-Glu levels (F_(1,114)_ = 34.670, *P* < 0.0001; Fig. 2e), while the injection of L-Asp, at the same dose, did not change the extracellular concentration of L-Glu, compared to vehicle treatment (F_(1,120)_ = 0.281, *P* = 0.5969; Fig. 2f).

Finally, we evaluated whether D-Asp could evoke L-Glu release also in another brain region, such as the striatum. Differently from the PFC, we found that the injection of 500 mg/kg D-Asp in freely moving mice failed to increase extracellular levels of L-Glu in the striatum (F_(1,108)_ = 0.020, *P* = 0.8888; Fig. 2g). Similar to D-Asp, also L-Asp administration was unable to affect the release of striatal L-Glu (F_(1,96)_ = 0.0157, *P* = 0.9005; Fig. 2h).

### Olanzapine, but not clozapine, inhibits murine and human D-aspartate oxidase activity

Based on the evidence that DDO plays a pivotal role in the regulation of D-Asp content in the mammalian brain^13^ and considering the reduction of the activity of this enzyme detected in patients with schizophrenia^18^, here we screened the potential ability of wide spectrum drugs with psychiatric relevance to affect the human DDO (hDDO) activity. To this aim, we performed *in vitro* inhibition assays on recombinant hDDO enzyme, using first-generation (chlorpromazine and haloperidol) and second-generation (clozapine and olanzapine) antipsychotics, and antidepressants (amitriptyline, bupropion and fluoxetine) (Table 1). In the reaction mixtures, different concentrations of drugs (in the 0–1000 μM range) were added to the same amount of hDDO (0.17 U) at a physiological concentration of exogenous FAD (4 μM). Noteworthy, biochemical data revealed that olanzapine was the only drug effective in regulating hDDO *in vitro*, by significantly reducing its catabolic activity (Table 1).

**Figure 3:**
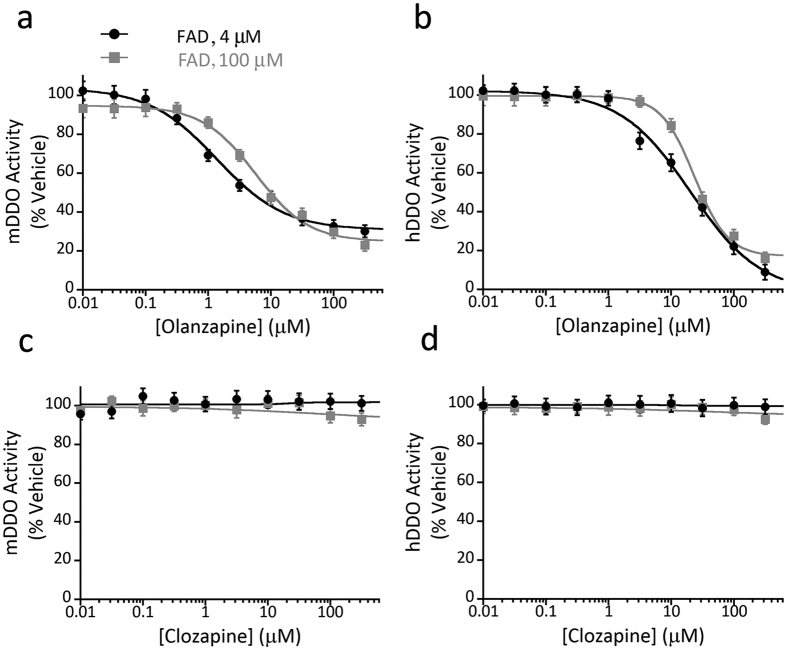
Olanzapine inhibits murine and human D-aspartate oxidase activity. (**a**–**d**) Enzyme inhibition assays performed by using recombinant (**a**,**c**) mouse DDO (mDDO) or (**b**,**d**) human DDO (hDDO) and (**a**,**b**) olanzapine or (**c**,**d**) clozapine as potential inhibitors. DDO activity in the presence of different concentrations of the antipsychotics (in the 0–300 μM range) and two FAD concentrations (4 and 100 μM) was determined by the Amplex UltraRed assay and an automated liquid handler system. Olanzapine significantly inactivates both mDDO and hDDO, whereas the activity of the two enzymes is unaffected by clozapine. The plots display the mean values ± SD, n = 3.

Before assessing whether olanzapine could affect DDO also in living mice, we preliminary tested *in vitro* the efficacy of this second-generation antipsychotic in inhibiting the recombinant mouse DDO (mDDO) activity. In this experiment, we used as a negative control another second-generation antipsychotic, clozapine, which was ineffective against hDDO (Table 1). Moreover, to evaluate whether the observed effect of olanzapine was due to the low concentration of FAD cofactor in the reaction mixture, we repeated the inhibition assays on both recombinant hDDO and mDDO using either a saturating (100 μM) or a lower, physiological (4 μM) concentration of exogenous FAD. A similar specific inhibition effect exerted by olanzapine was evident in both the conditions tested on the human and the murine enzyme. Indeed, at increasing concentrations of olanzapine, DDO activity decreased according to a classical sigmoidal dose-response curve (Fig. 3a,b). The regression equation allowed to estimate an IC50 value in the low micromolar range (mDDO: 5.6 ± 0.8 μM, hDDO: 23.4 ± 1.6 μM). Notably, using the lower concentration of FAD (4 μM) in the assay mixtures, a 4-fold increase of the potency of olanzapine on mDDO was observed (IC50 = 1.4 ± 0.2 μM; Fig. 3a), whereas the potency on hDDO was not affected (IC50 = 23.1 ± 3.2 μM, Fig. 3b). On the other hand, at both FAD concentrations (4 and 100 μM), the enzymatic activity of both mDDO and hDDO was unaffected by clozapine at all concentrations tested (Fig. 3c,d).

### Olanzapine triggers D-aspartate and L-glutamate release in the prefrontal cortex of freely moving mice

**Figure 4:**
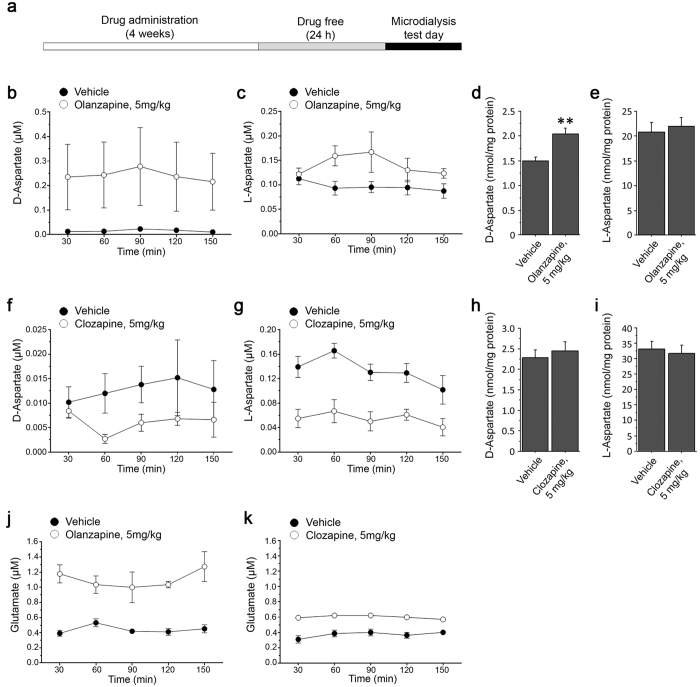
Chronic administration of olanzapine, but not clozapine, triggers the increase of extracellular D-aspartate and L-glutamate in the prefrontal cortex of mice. (**a**) Schematic timeline of the chronic antipsychotic drugs administration procedure and microdialysis. (**b**,**c**) Time course of free (**b**) D-Asp and (**c**) L-Asp extracellular concentration in the prefrontal cortex (PFC) of mice chronically treated with 5 mg/kg olanzapine and their relative vehicle-treated controls (n = 8 vehicle, n = 5 olanzapine). (**d**) Free D-Asp and (**e**) L-Asp total content in PFC homogenates of chronically olanzapine-treated mice and their relative vehicle-treated controls (n = 5 vehicle, n = 7 olanzapine). (**f**,**g**) Time course of free (**f**) D-Asp and (**g**) L-Asp extracellular concentration in the PFC of mice chronically treated with 5 mg/kg clozapine and their relative vehicle-treated controls (n = 5 per treatment). (**h**) Free D-Asp and (**i**) L-Asp total content in PFC homogenates of chronically clozapine-treated mice and their relative vehicle-treated controls (n = 10 per treatment). The amount of free D-Asp and L-Asp in tissue homogenates was normalized by the total protein content of each sample. (**j**,**k**) Time course of free L-Glu extracellular concentration in mice subjected to the chronic administration of 5 mg/kg (**j**) olanzapine or (**k**) clozapine and their relative vehicle-treated controls. We used the same cohorts of animals to concomitantly detect D-Asp, L-Asp and L-Glu. The graphs display the mean values ± SEM. **P < 0.01, compared to vehicle-treated mice (Student’s t test).

Based on the efficacy of olanzapine to affect the degradation of D-Asp *in vitro* through the inhibition of DDO, we explored whether this second-generation antipsychotic could affect the levels of this D-amino acid also *in vivo*. To this aim, we analyzed the extracellular levels of D-Asp in the PFC of olanzapine-treated freely moving mice. Animals were daily treated i.p. with 5 mg/kg olanzapine or vehicle for 4 weeks, and then implanted with microdialysis probe in the PFC. Dialysates were collected in drug-free condition, 24 h after the last injection (Fig. 4a). In parallel, we performed the same experiments in mice treated with clozapine (5 mg/kg), used as a negative control. Interestingly, the analysis of the dialysates revealed that the administration of 5 mg/kg olanzapine induced a significant increase of D-Asp efflux, compared to vehicle administration (two-way ANOVA with repeated measures: F_(1,11)_ = 5.037, *P* = 0.0463; average values: vehicle vs. olanzapine, 0.016 ± 0.002 vs. 0.242 ± 0.010 μM; Fig. 4b). Similarly, we found elevated free L-Asp levels in the PFC of olanzapine-treated mice, compared to vehicle-treated littermates (F_(1,11)_ = 7.059; *P* = 0.0223; average values: vehicle vs. olanzapine, 0.097 ± 0.004 vs 0.140 ± 0.009 μM; Fig. 4c). Consistent with the higher extracellular levels of D-Asp, we also detected a significant increase in the content of this D-amino acid in PFC homogenates of olanzapine-treated animals (vehicle vs. olanzapine: 1.50 ± 0.08 vs. 2.04 ± 0.12 nmol/mg protein; *P* = 0.0064; Fig. 4d). On the other hand, the total content of L-Asp was unaffected by the administration of this antipsychotic (vehicle vs. olanzapine: 20.83 ± 2.00 vs. 22.05 ± 1.59 nmol/mg protein; *P* = 0.6419; Fig. 4e). Differently to olanzepine, we found that chronic administration of 5 mg/kg clozapine did not significantly alter cortical extracellular levels of D-Asp (F_(1,32)_ = 3.141, *P* = 0.1143; average values: vehicle vs. clozapine, 13 ± 2 vs. 6.0 ± 1.0 nM; Fig. 4f), while it significantly decreased L-Asp efflux (F_(1,32)_ = 24.167, P = 0.0012; average values: vehicle vs. clozapine, 133 ± 1.0 vs. 55.0 ± 0.6 nM; Fig. 4g). Tissue homogenates analysis showed that clozapine administration did not induce significant changes of both total D-Asp (vehicle vs. clozapine: 2.29 ± 0.19 vs. 2.46 ± 0.22 nmol/mg protein; *P* = 0.5622; Fig. 4h) and L-Asp content (vehicle vs. clozapine: 33.22 ± 2.49 vs. 31.67 ± 2.65 nmol/mg protein; *P* = 0.6753; Fig. 4i).

Besides their well-known effect on dopamine and serotonin neurotransmission, some atypical antipsychotics can also influence the glutamatergic system^19^,^20^,^21^,^22^. Here, we assessed whether chronic olanzapine and clozapine administration induce L-Glu efflux in the mouse PFC. Neurochemical analysis revealed increased cortical extracellular levels of L-Glu in olanzapine- and clozapine-treated freely moving mice, compared to their respective vehicle-treated groups (F_(1,11)_ = 49.530, *P* < 0.0001; average values: vehicle vs. olanzapine, 0.441 ± 0.024 vs. 1.104 ± 0.052 μM; Fig. 4j; F_(1,32)_ = 142.147, P < 0.0001; average values: vehicle vs. clozapine 0.37 ± 0.01 vs. 0.60 ± 0.01 μM; Fig. 4k).

### Olanzapine-mediated L-glutamate release is abolished in the prefrontal cortex of *D-aspartate oxidase* knockout mice

**Figure 5:**
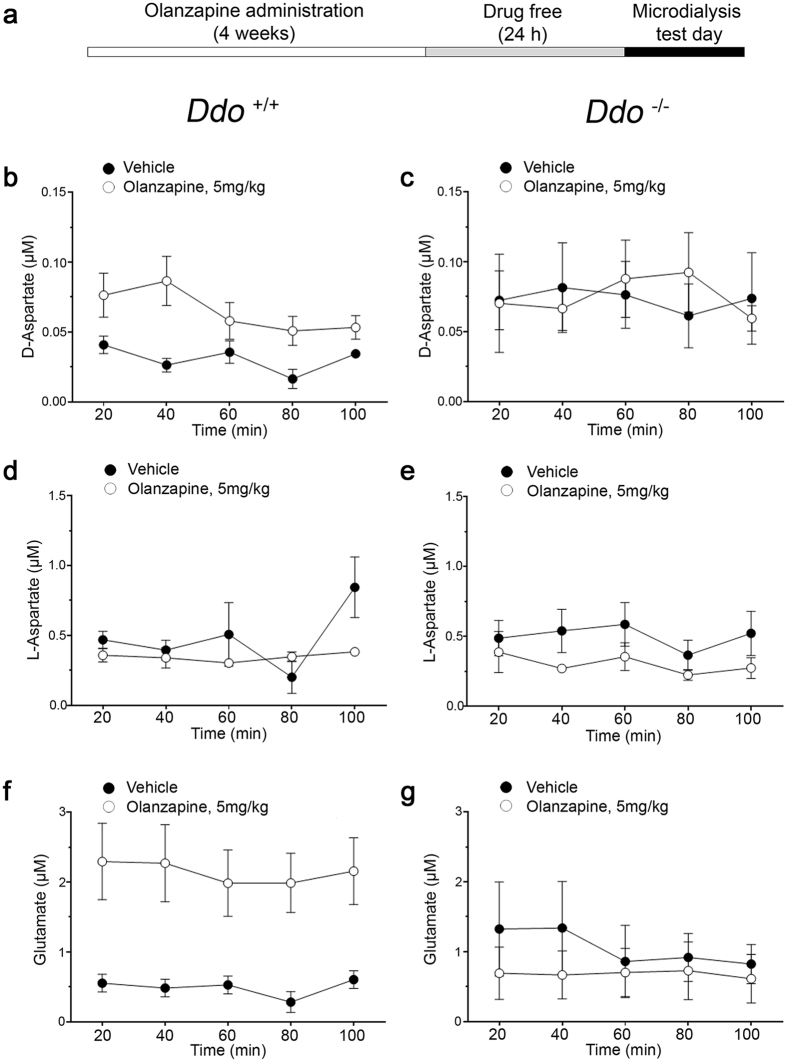
Olanzapine-mediated D-aspartate and L-glutamate release is abolished in the prefrontal cortex of *D-aspartate oxidase* knockout mice. (**a**) Schematic timeline of chronic olanzapine administration procedure and of the microdialysis experiments. (**b**–**g**) Time course of free (**b**,**c**) D-Asp, (**d**,**e**) L-Asp and (**f**,**g**) L-Glu extracellular concentrations in the prefrontal cortex of (**b**,**d**,**f**) *Ddo*^+/+^ (n = 4 vehicle, n = 7 olanzapine) and (**c**,**e**,**g**) *Ddo*^−/−^ mice (n = 5 vehicle, n = 7 olanzapine) chronically treated with 5 mg/kg olanzapine and their relative vehicle-treated controls. The graphs display the mean values ± SEM.

Considering the ability of olanzapine to inhibit *in vitro* the DDO activity, we analyzed whether, and to what extent, the release of cortical D-Asp and L-Glu triggered by this antipsychotic is mediated by DDO enzymatic inhibition also *in vivo*. To answer this question, we performed microdialysis experiments in freely moving mice lacking DDO (*Ddo*^−/−^) and their relative controls (*Ddo*^+/+^) chronically treated with olanzapine or vehicle (Fig. 5a). Two-way ANOVA with repeated measures confirmed a significant increase of the extracellular D-Asp levels in *Ddo*^+/+^ mice chronically treated with 5 mg/kg olanzapine (F_(1,9)_ = 9.576, *P* = 0.0128; average values: vehicle vs. olanzapine, 0.031 ± 0.004 vs. 0.065 ± 0.007 μM, Fig. 5b). On the other hand, olanzapine was ineffective in *Ddo*^−/−^ mice (F_(1,10)_ = 0.004, *P* = 0.9479; average values: vehicle vs. olanzapine, 0.073 ± 0.003 vs. 0.075 ± 0.006 μM, Fig. 5c). Moreover, statistical analysis revealed unchanged L-Asp levels in olanzapine-treated animals of both genotypes (*Ddo*^+/+^: F_(1,9)_ = 1.779, *P* = 0.2151; average values: vehicle vs. olanzapine, 0.484 ± 0.105 vs. 0.346 ± 0.013 μM; *Ddo*^−/−^: F_(1,10)_ = 2.718, *P* = 0.1302; average values: vehicle vs. olanzapine, 0.501 ± 0.037 vs. 0.303 ± 0.030 μM; Fig. 5d,e). Importantly, in line to D-Asp, cortical extracellular levels of L-Glu were significantly increased in olanzapine-treated *Ddo*^+/+^ but not in *Ddo*^−/−^ mice (*Ddo*^+/+^: F_(1,9)_ = 8.585, *P* = 0.0168; average values: vehicle vs. olanzapine, 0.490 ± 0.056 vs. 2.139 ± 0.066 μM; *Ddo*^−/−^: F_(1,10)_ = 0.414, *P* = 0.5346; average values: vehicle vs. olanzapine, 1.053 ± 0.115 vs. 0.681 ± 0.019 μM; Fig. 5f,g).

## Discussion

A large bulk of studies suggests that D-Asp has a functional role in endocrine glands where it modulates the synthesis and release of different hormones, including testosterone^23^,^24^. On the other hand, the involvement of this D-amino acid in brain physiology is still largely unknown. Here we reported that acute injection of D-Asp rapidly enhances its extracellular levels in the PFC of freely moving mice, suggesting that exogenous D-Asp can efficiently cross the blood brain barrier, as described so far only for D-serine and D-proline^25^,^26^. The evidence of D-Asp influx into the brain parenchyma nicely explains our previous result showing that acute injection of D-Asp, at the same dose tested here, significantly increases the cerebellar levels of cGMP^9^, which is regarded as a reliable *in vivo* index of NMDAR activity^27^. Moreover, we observed that acute D-Asp administration also elicits a transient increase of extracellular L-Asp, without affecting the amount of this L-amino acid in homogenates. To explain this data, we suggest that abnormal accumulation of D-Asp in the brain may perturb one or more of the still unclear cellular mechanisms regulating the local racemization of D-Asp into L-Asp^28^,^29^, L-Asp vesicular storage^30^,^31^ and release^32^. In addition, we cannot exclude that non-physiological, high D-Asp extracellular concentration may displace L-Asp from its binding to L-Glu/L-Asp transporters, which regulate the uptake of both Asp enantiomers^33^, thus resulting in increased extracellular L-Asp levels.

Like acute treatment, chronic oral administration of D-Asp enhances its extracellular levels in the mouse PFC, further confirming the efficiency of this D-amino acid to cross the blood brain barrier. Moreover, in line with recent microdialysis studies^4^, we showed that the *in vivo* release of D-Asp occurs through Ca^2+^-dependent processes, since its levels rapidly decrease below detection limits in Ca^2+^-free dialysates. The fast clearance of D-Asp upon Ca^2+^ depletion also indicates that this D-amino acid is actively removed from the extracellular *milieu*, most likely through the L-Glu/L-Asp transport system^33^. Future studies are needed to unravel the mechanisms and the cell types involved in D-Asp release and uptake *in vivo*. Unlike acute D-Asp injection, chronic administration of D-Asp did not produce any change in L-Asp release in the mouse PFC. To explain this apparent discrepancy, we hypothesize that the diverse magnitudes of D-Asp changes evoked by the two protocols and/or the duration of the increase itself (transient vs. chronic) may differently affect the L-Glu/L-Asp transport system and/or the local mechanisms of L-Asp release.

Here, we also confirmed that systemic treatment with D-Asp triggers the *in vivo* efflux of L-Glu in the mouse PFC. Interestingly, the elevation of both extracellular D-Asp and L-Glu levels in orally D-Asp-treated mice mirrors the greater NMDAR-dependent miniature excitatory post-synaptic currents and *in vivo* cerebral activity measured by fMRI in the PFC of mice under this regimen of D-Asp administration^11^. To explain the mechanism of action underpinning the cortical release of L-Glu in D-Asp-treated mice, we assessed whether D-Asp could influence the presynaptic NMDA and non NMDA receptors responsible for L-Glu efflux^34^,^35^ in synaptosomal preparations from mouse PFC. Notably, we found that D-Asp stimulates presynaptic L-Glu release acting on NMDA, AMPA/kainate and mGlu5 receptors. This observation fits with the presence of facilitatory NMDA and non-NMDA ionotropic and metabotropic autoreceptors on cerebrocortical glutamatergic nerve terminals^36^,^37^,^38^,^39^. Interestingly, NMDA autoreceptors have not been found on cortico-striatal or thalamo-striatal glutamatergic terminals, suggesting that the presence of different populations of presynaptic receptors on cortical and striatal nerve terminals could explain the inability of D-Asp to evoke L-Glu release in the striatum of freely moving animals. Remarkably, we also found that the *in vivo* release of L-Glu is a stereoselective process since intraperitoneal L-Asp injection, at the same dose used for the D-enantiomer, failed to stimulate the efflux of this neurotransmitter in mice. Consistent with this evidence and considering that both D-Asp and L-Asp are able to stimulate L-Glu release in the synaptosomal preparation, we argue that the different *in vivo* effect of the two enantiomers in the mouse PFC may depend on their different efficiency in crossing the blood brain barrier. Future studies are requested to shed light on this important physiological issue, not yet explained at molecular level.

Besides the *in vitro* effect produced by D-Asp on presynaptic terminals, synaptosomal studies revealed that L-Asp stimulation was insensitive to subtype selective L-Glu receptor antagonists. This is consistent with a study on hippocampal slices where D-Asp but not L-Asp was found to bind to AMPA/kainate receptors^40^. Conversely, the insensitivity of L-Asp to MK801 is puzzling in view of the fact that L-Asp has been shown to bind^41^ and stimulate^42^ NMDAR, with K_d_ (11 μM) and EC50 (17 μM) values close to the concentration used in the present study. It is possible that cortical presynaptic NMDAR belongs to a subpopulation with low sensitivity to L-Asp, since presynaptic NMDAR may differ in subunit composition^43^. Differently from the present study, both D-Asp and L-Asp have been reported to stimulate NMDA and non-NMDA ionotropic, and group I metabotropic receptors in *substantia nigra* slices^44^. However, in that study the effect of endogenous L-Glu, released either directly via presynaptic receptors or indirectly through uptake reversal, could not be dissected out. Conversely, the continuous perfusion and the presence of the EAAT blocker TBOA in our synaptosomes preparations, make the contribution of extracellular L-Glu to receptor stimulation negligible.

The results reported above provide new evidence about the physiological mechanisms by which D-Asp influences glutamatergic transmission and, in turn, suggest a rationale to explore whether this D-amino acid may contribute to regulate the effect of second-generation antipsychotics on glutamatergic system. Indeed, previous findings indicate that olanzapine and clozapine, besides their effect on blockade of serotonin 5-HT2A receptors, direct or indirect stimulation of 5-HT1A receptors and, to a lesser extent, reduction of dopamine D2 receptor-mediated neurotransmission^45^, also affect glutamatergic system in humans^19^,^22^ and rats^20^,^21^. Interestingly, we reported that both antipsychotics increase extracellular L-Glu levels in the PFC of chronically treated mice. Besides their common influence on cortical L-Glu release, we found that olanzapine, but not clozapine, perturbs D-Asp metabolism in the mouse PFC *in vivo*. This finding is mechanistically substantiated by the peculiar ability of olanzapine to significantly inhibit hDDO and mDDO activity *in vitro*. In this regard, we reported that olanzapine was more potent towards hDDO than mDDO. In the absence of details that can be provided by the resolution of the protein structure only, this observation is possibly explained by the different fine modulation exerted by non-conserved residues in the active site, as well as in the cofactor binding domain of the two homologous DDOs, similar to that previously reported for rat vs. human D-amino acid oxidase^46^. Noteworthy, the inhibitory effect of olanzapine on recombinant hDDO is only restricted to this compound since it is not replicated with other psychiatric medications, including antidepressants (amitriptyline, bupropion and fluoxetine) and first-generation antipsychotics (chlorpromazine and haloperidol). Also clozapine has proven to be ineffective in suppressing DDO activity, further indicating the existence of different pharmacological targets for these two widely used second-generation antipsychotics^20^,^47^. On the other hand, the reason by which clozapine significantly reduces the cortical L-Asp release in treated animals remains unknown and requires future detailed investigations. Taken together, the present data suggest that the increased DDO activity and the resulting decrease in D-Asp levels recently found in the *post-mortem* brain of patients with schizophrenia^18^ are not an epiphenomenon of pharmacological treatment, at least in the context of the antipsychotics used here. Remarkably, for the first time, our observations put forward a translational value for D-Asp metabolism since they highlight an unexpected instrumental role for DDO activity in the modulation of glutamatergic effects of olanzapine. However, since DDO has intracellular localization and is active within peroxisomes^13^, future studies are needed to clarify whether and how olanzapine has direct access to DDO *in vivo*. Regardless of the precise mechanism responsible for DDO inhibition, the potential relevance of DDO as a pharmacological target in schizophrenia shown by this and previous finding^18^ prompts to the discovery of novel compounds with inhibitory activity for this enzyme, as recently reported by Homma and co-workers^48^. In agreement with its agonistic profile not only at NMDARs but also at mGlu5 receptors^49^, previous observations showed that increased levels of D-Asp in preclinical models are associated with improved memory^6^,^7^,^9^, enhanced structural and functional synaptic plasticity^11^ and greater connectivity in cortico-hippocampal regions^16^. Thus, considering that supplementation of D-Asp proved to be safe even at high doses in humans^50^,^51^, we hypothesize that the add-on of this D-amino acid to second-generation antipsychotics could disclose beneficial effects in the treatment of cognitive deficits in schizophrenia. Nevertheless, future clinical studies investigating the link between D-Asp metabolism and olanzapine are warranted to confirm also in humans the present observations and, in turn, to find novel therapeutic avenues for treating neuropsychiatric disorders associated to hypofunctional cortical glutamatergic neurotransmission.

## Methods

### Animals

Three/four-month-old C57BL/6J male mice were purchased from Jackson Laboratory (Bar Harbour, ME, USA). Knockout male mice for the *Ddo* gene were generated and genotyped by PCR as described previously^52^. Animals were group housed (five per cage), at a constant temperature (22 ± 1 °C) on a 12 h light/dark cycle (lights on at 7 AM) with food and water *ad libitum*. All research involving animals was carried out in accordance with the Italian directive of the Ministry of Health governing animal welfare and protection (D.LGS26/2014), and approved by “Direzione Generale della Sanità e dei Farmaci Veterinari (Ufficio 6)” (permission nr 1134/2016).

### Treatments

C57BL/6J mice were chronically administered with D-Asp for one month by delivering the D-amino acid (20 mM) in drinking water, or acutely administered by intra-peritoneal (i.p.) injection (500 mg/kg D-Asp, dissolved in 0.9% NaCl). Five mg/kg olanzapine, 5 mg/kg clozapine and their respective vehicle solutions were i.p. injected once a day for 4 weeks to C57BL/6J, *Ddo*^+/+^ and *Ddo*^−/−^ mice. Olanzapine was dissolved in saline solution. Clozapine was dissolved in a minimum amount of 0.1 N HCl and then diluted with saline (adjusted to pH 6–7 with 0.1 N NaOH)^53^. Chronic treatments with D-Asp, olanzapine or clozapine were interrupted 24 h before collection of perfusates by microdialysis.

### *In vivo* microdialysis

Microdialysis experiments were performed in awake and freely moving mice^4^,^54^. C57BL/6J mice were anaesthetized (50 mg/kg pentobarbital, i.p.) and stereotaxically implanted with microdialysis probes into the PFC (see Supplementary Methods for details). After a 24-h post-operative recovery, probes were perfused at 1 μL/min with artificial cerebrospinal fluid (ACSF: 147 mM NaCl, 2.2 mM CaCl_2_, 4 mM KCl; pH 7.2) by a Harvard Apparatus (Holliston, MA, USA) infusion pump. After a 60-min equilibration period, dialysates were collected every 20 or 30 min (for acute or chronic administration, respectively), and then analyzed for amino acid content. Samples collected in the absence of calcium were obtained by perfusion with Ca^2+^-free ACSF solution.

### HPLC analysis

D-Asp, L-Asp and L-Glu content in dialysates was analyzed by HPLC as previously reported^4^ (see Supplementary Methods for further details). D-Asp and L-Asp content in PFC homogenates was measured as previously reported^55^, with minor modifications. (see Supplementary Methods for further details). Statistical analysis of amino acids extracellular levels was performed by two-way ANOVA with repeated measures (treatment × time). Statistical analysis of amino acids levels in homogenates was performed by the Student’s t test.

### Synaptosomes preparation

Synaptosomes were isolated from the mouse frontal cortex as previously described^14^,^56^. The synaptosomal pellet was resuspended in oxygenated (95% O_2_, 5% CO_2_) Krebs solution (mM: NaCl 118.5, KCl 4.7, CaCl_2_ 1.2, MgSO_4_ 1.2, KH_2_PO_4_ 1.2, NaHCO_3_ 25, glucose 10) and perfused as described in Supplementary Methods. L-Glu levels in the synaptosome perfusate were measured by HPLC coupled to fluorimetric detection, as previously described^57^.

### Inhibition assays

The effect of antidepressant and antipsychotic drugs on recombinant hDDO and mDDO activity was evaluated *in vitro* using a coupled enzyme assay and the Amplex UltraRed reagent (Invitrogen, ThermoFisher Scientific, Waltham, MA, 0245, USA)^58^,^59^ (see Supplementary Methods for further details). Briefly, 0.17 U/ml of hDDO or mDDO and 0.1 U/ml horseradish peroxidase (Roche, Basel, Switzerland) were incubated for 30 min with the different drugs (0–1000 μM concentration range) in the presence of exogenous FAD (4 or 100 μM). Then, D-Asp (7.5 mM) and the Amplex UltraRed reagent (35 μM) were added and the fluorescence of oxidized reagent produced by DDO activity was measured after 30 min (540 and 595 nm: excitation and emission wavelengths, respectively). Further procedure is described in Supplementary Methods.

## Additional Information

**How to cite this article**: Sacchi, S. *et al*. Olanzapine, but not clozapine, increases glutamate release in the prefrontal cortex of freely moving mice by inhibiting D-aspartate oxidase activity. *Sci. Rep.* **7**, 46288; doi: 10.1038/srep46288 (2017).

**Publisher's note:** Springer Nature remains neutral with regard to jurisdictional claims in published maps and institutional affiliations.

## Supplementary Material

Supplementary Information

## Figures and Tables

**Table 1:** Inhibition assays showing the relative activity (%) of recombinant human DDO in the presence of different concentrations (1–1000 μM range) of first- (chlorpromazine, haloperidol) and second-generation antipsychotic (clozapine, olanzapine), and antidepressant drugs (amitriptyline, bupropion, fluoxetine).

Drug	Chemical structure	Concentration (μM)			
		1	10	100	1000
Chlorpromazine	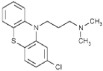	100	100	100	>90
Haloperidol	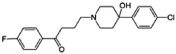	>95	>95	100	100
Clozapine	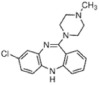	100	100	>95	>95
Olanzapine	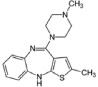	97	81	10.4	2
Amitriptyline	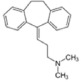	100	100	100	100
Bupropion	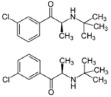	>95	93	>95	89
Fluoxetine	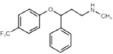	100	>95	>95	100
