# Supplementary material for: Olanzapine, but not clozapine, increases glutamate release in the prefrontal cortex of freely moving mice by inhibiting D-aspartate oxidase activity
Source: Sci Rep. 2017 Apr 10;7:46288. doi: 10.1038/srep46288 (PMC5385520; doi:10.1038/srep46288)
Supplement: Supplementary Information [file srep46288-s1.docx]

**Supplementary Information**

Olanzapine, but not clozapine, increases glutamate release in the prefrontal cortex of freely moving mice by inhibiting D-aspartate oxidase activity

Silvia Sacchi^1,2,^*, Vito De Novellis^3,^*, Giovanna Paolone^4,^*, Tommaso Nuzzo^5,6^, Monica Iannotta^3^, Carmela Belardo^3^, Marta Squillace^5^, Paolo Bolognesi^4^, Elena Rosini^1,2^, Zoraide Motta^1^, Martina Frassineti^4^, Alessandro Bertolino^7^, Loredano Pollegioni^1,2^, Michele Morari^4^, Sabatino Maione^3^, Francesco Errico^5,8, @^, Alessandro Usiello^5,6,@^

^1^Dipartimento di Biotecnologie e Scienze della Vita, Università degli studi dell’Insubria, 21100, Varese, Italy; ^2^The Protein Factory, Politecnico di Milano and Università degli studi dell’Insubria, 20131, Milano, Italy; ^3^Department of Experimental Medicine, Section of Pharmacology, The Second University of Naples (SUN), 80138, Naples, Italy; ^4^Department of Medical Sciences, Section of Pharmacology, University of Ferrara and National Institute of Neuroscience, 44100, Ferrara, Italy; ^5^Laboratory of Behavioural Neuroscience, Ceinge Biotecnologie Avanzate, 80145, Naples, Italy; ^6^Department of Environmental, Biological and Pharmaceutical Sciences and Technologies, Second University of Naples (SUN), 81100, Caserta, Italy; ^7^Department of Basic Medical Science, Neuroscience and Sense Organs, University of Bari Aldo Moro, 70121, Bari, Italy; ^8^Department of Molecular Medicine and Medical Biotechnology, University of Naples “Federico II”, 80131, Naples, Italy.

**Supplementary Methods**

*Drugs and chemicals*

N-Acetyl-L-cysteine (NAC), *o*-phtaldialdehyde (OPA), D-Asp, L-Asp, clozapine, serum albumin and DMSO, were from Sigma-Aldrich (St. Luis, MO, USA); olanzapine was from Zyprexa, Eli Lilly and Co. (Indianapolis, IN, USA).

*In vivo microdialysis*

Stereotaxic implant into the PFC was performed using the following coordinates AP: 1.8 mm; L: 0.4 mm from bregma and V: 3.0 mm below the dura (see Figure 1A), and secured to the skull using stainless steel screws and dental cement. Microdialysis probes were constructed with 22G (0.41 mm I.D., 0.7 mm O.D.) stainless steel tubing: inlet and outlet cannulae (0.04 mm I.D., 0.14 mm O.D.) consisted of fused silica tubing. The probe had a tubular dialysis membrane (Enka AG, Wuppertal, Germany) 1.3 mm in length.

*HPLC analysis*

The HPLC system used for dialysates analysis comprised a Varian ternary pump (mod. 9010), a C18 reverse-phase column, a Varian refrigerated autoinjector (mod. 9100) and a Varian fluorimetric detector. The mobile phase consisted of 2 components: a) 0.2 M sodium phosphate buffer pH 5.8, 0.1 M citric acid, and b) 90% acetonitrile, 10% distilled water. Dialysates were precolumn derivatized with OPA/NAC (in 50% methanol) and amino acid conjugates resolved using a gradient separation. To determine the peak area corresponding to D-Asp, selected samples were incubated in parallel with 20 µg of recombinant beef DDO for 15 min at 37 °C and analyzed as above. The disappearance/reduction of the area of D-Asp elution peak confirmed the presence of D-Asp and gave the exact D-Asp content. A typical analysis is depicted in Figure 1B. Data were collected by a Dell Corporation PC system 310 interfaced by Varian Star 6.2 control data and acquisition software.

For detection of D-Asp and L-Asp in PFC homogenates, we used an HPLC system consisting of a Jasco apparatus (Jasco-Europe, Cremella, Italy) equipped with a FP-2020 Plus fluorescence detector and a Symmetry C8 5 µm, 4.6x250 mm column (Waters, Milford, MA, USA). Mice were sacrificed, the PFC dissected out within 20 s on an ice-cold surface and then homogenized in 1:20 (w/v) 0.2 M TCA, sonicated (3 cycles, 10 s each) and centrifuged at 10000 g for 10 min. The supernatants were neutralized with NaOH, derivatized with OPA/NAC (10 mg/mL OPA and 5 mg/mL NAC in 50% methanol) and analyzed. Identification and quantification of D-Asp and L-Asp was based on retention times and peak areas, compared with those associated with external standards. The identity of D-Asp and L-Asp peaks was confirmed either by the addition of internal standards, and by a pre-column treatment with RgDAAO M213R variant (which is active on acidic D-amino acids) [^1^](#_ENREF_1) and StLASPO (active on L-Asp) [^2^](#_ENREF_2), respectively: the samples were added with 10 µg of the enzymes, incubated at 30 °C for 30 min and then derivatized. Calibration curves were built by injecting increasing amount of standards (0.25-50 pmol). Total protein content of PFC homogenates was determined by using the Bradford assay method, after resolubilization of the TCA precipitated protein pellets. The detected D-Asp and L-Asp total concentration in homogenates was normalized by the total protein content and expressed as nmol/mg protein; amino acids extracellular levels were expressed as μM.

*Synaptosomes preparation*

After cortical synaptosome isolation, 1 ml aliquot of the suspension (~0.35 mg protein) was slowly injected into nylon syringe filters (outer diameter 13 mm, 0.45 µM pore size, internal volume ~100 µL; Teknokroma, Barcelona, Spain) connected to a peristaltic pump. Filters were maintained at 36.5 °C and superfused at a flow rate of 0.4 mL/min with a pre-oxygenated Krebs solution containing the excitatory amino acid transporter inhibitor DL-threo-β-benzyloxyaspartic acid (TBOA, 10 µM), to prevent L-Glu release from transport reversal. Sample collection (every 3 min) was initiated after a 20-min filter washout. The effects of D-Asp (10 µM), L-Asp (10 µM) and NMDA (10 µM) on 15 mM K^+^-stimulated (90 s pulse) neurotransmitter outflow were evaluated. NMDA and D-Asp concentrations were chosen based on concentration-response curves generated in previous studies [^3^](#_ENREF_3)^,^[^4^](#_ENREF_4). Agonists were added to the perfusion medium 3 min prior to K^+^ and maintained in the perfusion fluid for additional 3 min. The NMDAR antagonist MK801 (10 µM), the AMPA/kainate receptor antagonist CNQX (0.3 µM) and the mGlu5 receptor (mGluR5) negative allosteric modulator MTEP (0.3 µM) were perfused 3 min prior to agonists and maintained until the end of experiment.

*Enzymes and inhibition assays*

Recombinant M213R variant of *Rhodotorula gracilis* D-amino acid oxidase (RgDAAO; EC 1.4.3.3) and *Sulfolobus tokodaii* L-aspartate oxidase (StLASPO; EC 1.4.3.16) were overexpressed in *E. coli* cells and purified as previously described [^1^](#_ENREF_1)^,^[^2^](#_ENREF_2). The final M213R RgDAAO and StLASPO preparations had a specific activity of 5.8 U/mg protein on D-Asp and 0.98 U/mg protein on L-Asp, respectively. Recombinant DDO (EC 1.4.3.1) from beef kidney was expressed in *E. coli* and the purified preparation showed a specific activity of 5 U/mg protein on D-Asp [^5^](#_ENREF_5). These flavoenzymes were fully inactive on the opposite enantiomers. Recombinant D-aspartate oxidase from human (hDDO) and mouse (mDDO) origin were overexpressed in *E. coli* cells and purified as previously reported [^6^](#_ENREF_6), with minor modifications.

For inhibition assay, 10 mM chlorpromazine, haloperidol, olanzapine, clozapine, amitriptyline, bupropion and fluoxetine stock solutions were prepared by dissolving the drugs in DMSO, and subsequently diluted in 50 mM sodium phosphate, pH 7.4. Vehicle concentration was carefully maintained such that all experimental groups had the same DMSO concentration (never exceeding 1.5%). After incubation of recombinant hDDO or mDDO with the different drugs (0-1000 µM), the fluorescence of oxidized Amplex UltraRed produced by DDO activity was measured in endpoint mode (540 and 595 nm as excitation and emission wavelengths, respectively). All enzymatic assays were conducted at room temperature in 96-well plate format using an automated liquid-handler system (epMotion 5075; Eppendorf, Hamburg, Germany). Data were fit to a standard, four parameters equation to determine curve top, bottom, concentration producing 50% inhibition (IC_50_) and Hill slope [^7^](#_ENREF_7)^,^[^8^](#_ENREF_8).

**Supplementary References**

1 Sacchi, S. *et al.* Engineering the substrate specificity of D-amino-acid oxidase. *The Journal of biological chemistry* **277**, 27510-27516, doi:10.1074/jbc.M203946200 (2002).

2 Bifulco, D., Pollegioni, L., Tessaro, D., Servi, S. & Molla, G. A thermostable L-aspartate oxidase: a new tool for biotechnological applications. *Applied microbiology and biotechnology* **97**, 7285-7295, doi:10.1007/s00253-013-4688-1 (2013).

3 Cristino, L. *et al.* d-Aspartate oxidase influences glutamatergic system homeostasis in mammalian brain. *Neurobiology of aging* **36**, 1890-1902, doi:10.1016/j.neurobiolaging.2015.02.003 (2015).

4 Marti, M. *et al.* Striatal glutamate release evoked in vivo by NMDA is dependent upon ongoing neuronal activity in the substantia nigra, endogenous striatal substance P and dopamine. *Journal of neurochemistry* **93**, 195-205, doi:10.1111/j.1471-4159.2005.03015.x (2005).

5 Negri, A., Tedeschi, G., Ceciliani, F. & Ronchi, S. Purification of beef kidney D-aspartate oxidase overexpressed in Escherichia coli and characterization of its redox potentials and oxidative activity towards agonists and antagonists of excitatory amino acid receptors. *Biochim Biophys Acta* **1431**, 212-222 (1999).

6 Katane, M. *et al.* Comparative characterization of three D-aspartate oxidases and one D-amino acid oxidase from Caenorhabditis elegans. *Chemistry & biodiversity* **7**, 1424-1434, doi:10.1002/cbdv.200900294 (2010).

7 Hopkins, S. C. *et al.* Structural, kinetic, and pharmacodynamic mechanisms of D-amino acid oxidase inhibition by small molecules. *Journal of medicinal chemistry* **56**, 3710-3724, doi:10.1021/jm4002583 (2013).

8 Terry-Lorenzo, R. T. *et al.* Novel human D-amino acid oxidase inhibitors stabilize an active-site lid-open conformation. *Bioscience reports* **34**, doi:10.1042/BSR20140071 (2014).
